# Editorial Department

**Published:** 1883-04

**Authors:** 


ELMIRA, N. Y., APRIL, 1883.

The BISTOURY is published Quarterly, upon the first of April, July, October and January, at Fifty Cents a year in advance.

### Editorial Department.

THAT DORSEY MATTER.—We do not know that the time will ever arrive when honest men will entirely get their rights, but it does seem that the season is redolent with dishonesty coming to grief, especially amongst those in high places. Bank Presidents, State Treasurers, Poor-house officials, Star Route contractors, political bosses are found now side by side with other thieves in the dock, and in spite of legal tricks of eminent counsel, paid from the stolen pile, they find their way to the penitentiary.

There used to be an understanding among thieves that they would be honest towards one another. An item in the evidence of the Star Route cases, now on trial, seems to indicate that some have fallen from grace in that particular. It appears that a check written by Dorsey, for $2,000, for a gambling debt, has somehow come into the case. We give below a statement said to have emanated from Berdell, a witness in the Star Route trial:

"Dorsey lost nearly all the money he had, and in a settlement with Belford gave him this $2,000 check, which was put in bank for collection by either Belford or one of the winners, to whom Belford transferred the check in settlement. Dorsey was shrewd, and charged his loss to 'mail,' and thus generously permitted Bosler, Brady, Vaile & Co. to share his loss."

The above statement of Berdell has thrown some light upon the transaction, and the interested parties are having a lively time among themselves.

Dorsey fires off a card of denial, as to persons and names, against the Press Agent, and simultaneous with the card is this amended statement: "A telegram to the Associated Press from Denver, Colorado, says it is learned 'from an authentic source' that in July, 1879, Judge J. B. Bissell, of Leadville, won $2,000 from S. W. Dorsey in a game of poker on a train going to Denver, for which he gave a check. This is evidently the check referred to by Berdell as having been given to 'J. B. B.,' and imputed to Congressman J. B. Bedford."

S. W. Dorsey is an ex-senator of the United States; and amongst his associates in the transactions for which he is being tried are men who have occupied high positions of trust under the Government.

Let the good work go on, and when crime is legally traced, let the punishment be in proportion to the trust.

---

WIGGINS—VENNOR.—There are false prophets in these days. At a great outlay our Government has provided means for giving notice of the approach of meterological disturbances, which are the result of causes easily detected by the comparison of instruments for that purpose, placed at selected points throughout the country. When these causes are of sufficient magnitude to produce general, and not merely local storms, then the prognostications from the Bureau at Washington are generally correct. Local disturbances frequently interfere, however, and then we say "Old Prob" has failed.

So far, we have science, but the false prophets *guess*, because a storm sometimes, or frequently occurs at the vernal equinox, or geese fly north or south before or behind a gale, or the woodchuck looks out of his hole, or does not look out of it, or the breast bone of a fowl is rough or smooth, or the little pig's tail curls to the left or right, or some other absurd thing, then we say they *guess*. Or perhaps they are smarter and unscrupulous, and care not what course they pursue to gain the end they seek—notoriety. Vennor flashed up, and his light went out before doing any harm. It has been reserved for "Wiggins" to catch the weak and trusting by his bombastic claims to a knowledge of nature's laws, and whilst we pen this, men who "go down to the sea in ships" are staying at home, kept from their labors by his evil prophecies, and the daily reference to him in the papers indicate the depth of the confidence the superstitious have placed in his warnings. Perhaps the lesson will result well, should not, unfortunately, a coincidence in the shape of a storm give some title of credence to his folly. We look to see engrafted in a coming dictionary of new words: "Wiggins—One who sets himself up as a prophet and comes to grief."

THE TARIFF.—Business in this country can be conducted profitably upon almost any *settled* government policy. The people of the United States have industrious habits and ingenuity enough to adapt themselves to almost any situation they may be placed in, but it is discouraging to arrange plans of business, invest capital in machinery, and enter into contracts based upon laws which are liable, at any session of Congress, to be repealed or changed in the interest of some party faction or lobby pressure. In this position have our manufacturers and business men been for the past winter, and even now, when Congress has adjourned, and a tariff bill been passed, not satisfying any one, business men are threatened with an entire revision next winter.

It seems to us that the time is fast approaching when the people will insist upon more honest labor and less politics at Washington.

---

"GIN MILLS."—This is the appellation given the fashionable tippling places, where gentlemen drink their cocktails and smoke fragrant and expensive cigars. At this day, their appointments and decorations are so magnificent as to attract the attention of those who admire the beautiful. "Green's," at Eighth and Chestnut streets, and "Steele's," at Broad and Chestnut streets, Philadelphia, are the most recent extravagant establishments of this nature. Hundreds of thousands of dollars have been expended in the embellishment of these two famous drinking places. The ceilings are of the most costly and exquisite frescos; the side walls decorated with exquisite hangings and with paintings of the masters; bronzes and sculptures rest upon artistically wrought pedestals and tables; crystal chandeliers lighted with electric lights, and pyramids of the finest and most costly glassware sparkle and glitter, to bewilder the eye. Mirrors of fabulous price, so arranged as to multiply the brilliant surroundings, fairly transfixes the beholder with wonder at the fairy scene. If you would visit the most beautiful in art decorations, in Philadelphia, visit the "gin mills."

---

AN UNUSUAL FUNERAL.—Recently, at the burial of a once distinguished physician and scientist, who sacrificed his splendid attainments for the love of drink and a life of vagabondage, his son summoned to his aid eight trusted friends and quietly buried the old man, delivering at the grave the following message, which we have been asked to publish:

"Here lies the refuse of a once living man, whose life, while upon earth, was a failure—a failure because he *willed* it so to be, or did not will it to be otherwise. The occurrence is such an unusual one as to be noteworthy. Let us consider it for a moment: He was a man who, by reason of his educational attainments and acquirements in the Arts and Sciences, was eminently qualified for improving the condition of his fellows; and, indeed, did so minister to them until he had attained forty years of age.

"At the early age of eighteen he graduated as Master of Arts, in Dickinson College, of Pennsylvania. Before he was quite twenty-one he was passed to the degree of Doctor of Medicine, in the University of Pennsylvania, when he immediately entered an unusually successful career as a physician and surgeon. Under the guidance of a Quaker father and the gentle influence of a noble, Christian mother, he made rapid strides in a profession which, in *his* day, had but few skillful followers. He rose quickly to fame as a surgeon; his services were sought far and wide; fame and wealth—the generally prized belongings to man—were his. Marrying at an early age, he settled in a prosperous community where his talents were recognized and amply rewarded. To the casual observer, nothing seemed lacking that would enhance the desirability of his surroundings, or contribute to his happiness.

Why, then, this collapse, this total wreck of a life so full of promise, so vigorous, healthful, energetic, intelligent; so capable of glorious achievements for himself and his dependent ones? Why should he have died among strangers, in a remote land, poor, penniless, and unregretted?

"Reflecting over his career, scanning the possibilities that *should* have been his, yet were not; tracing his downward course, until fame, wealth, pride, family, friends, *all* were forsaken and his whole being entombed in a carapace that permitted of no intrusion upon his finer feelings—his better nature—I am led to the conclusion that all this change in his character was brought about through the education and quickening of his worldly attributes—that fame among men should be the more readily attained—to the entire and complete neglect of those qualities designated the spiritual.

"Had he been surrounded by a better companionship; had he mingled more with spiritually-minded men, or those even who had a wholesome regard for the opinions and practices of refined and recognized society; had he made a patient endeavor (even under those adverse circumstances that unhappily were present) to build up and shield his home and family, perhaps a better, brighter light would have kindled within him, lighting the way to nobler thoughts and holier aspirations, when a life would not have stranded upon wholly desolate shores.

"But let us not judge the dead; his career is finished—beyond his or our ability to amend—seeming, to *our* circumscribed vision, a failure.

"This ended life, then, if it teaches anything, points undeviatingly to the fact here sadly yet sufficiently demonstrated, that no man, however intellectually endowed, or however securely surrounded and protected by the accomplishments and requirements of mankind, can be secure in the attainment of a longed-for immortality, without throwing out a chain here and there, the links of which shall point toward the Infinite, securing an anchorage somewhere near his domain.

"Let us hope that it is well with him whose body we now bury, breathing a prayer to the Father, beseeching him to be more merciful to him than he was to himself."

---

A PROJECTING MICROSCOPE.—Prof. D. S. Holman, Actuary of the Franklin Institute, of Philadelphia, has devised a new instrument which he names the Holman Lantern Microscope. This ingenious instrument is the result of many years of experiment, on the part of Prof. Holman, to devise a good projecting microscope. It is made by Zentmyer, which is sufficient guaranty for perfect workmanship. The instrument can be used as a microscope, as a stereopticon, and as a projecting microscope, into all of which it is readily converted by the simplest means. It is used with an oxy-hydrogen lamp, or with an ordinary kerosene lamp. We were shown in an admirable manner the circulation in the gills of a salamander, thrown into a four feet circle, upon a screen. The crystalization of different salts was also shown, by aid of the polariscope, exhibiting all the beautiful colors in the crystals. For class lecturing this is certainly an admirable lantern. Doubtless those so employed will be glad to know of this new and complete instrument.

Prof. Holman also exhibited the new contrivance named the phoneidoscope—from *pheno*, a sound, *eidos*, an image, *scopio*, I see—hence, "the image of sound I see." By means of this ingenious device a film of soap, spanned across a tube, was illuminated by means of a powerful condenser, and then an image of it projected upon the screen. Singing in the other end of the tube set the film to vibrating, which vibrations were seen upon the screen. Different bands of colors were produced that varied with the intensity of the sound. The experiment is exceedingly ingenious in method as well as in the implement employed, and is supposed to be a verification of the wave theory of light as well as of sound.

---

A NEW TELEPHONE.—We have had the privilege of examining the new long distance telephone invented by Dr. Leslie Baxter, of Washington, D. C. Aside from its qualities as a long distance telephone—it having been successfully used between Washington and Cleveland—is its wonderful speaking qualities. It is not necessary to speak into the transmitter, nor is it required to hold the instrument to your ear. You can sit in your chair, anywhere in the room where the instruments are, and hold conversation as easily with a friend in a distant city as though he were in the room with you. We heard Dr. Baxter speaking, when thirty-two feet from the transmitter, quite as distinctly as when close to the instrument. A whisper is as easily heard as a shout. It is by all odds the plainest speaking telephone yet invented, and must supersede all other instruments of the kind. A company with one million dollars capital has been organized in New York, who will speedily introduce the new telephone to the public.

---

BUYING UP MANUFACTURING CONCERNS.—Granted that the more manufactories a city contains the larger number of people find employment, who in turn benefit the business interests of the city by extending its population that expends its earnings to build up the traffic of the place. But just whether it pays to expend thousands of dollars to *induce* established manufactories in neighboring towns to locate among us, is admissable of debate. Private citizens of this city have raised a purse of $20,000 to build shops for steam engine works that have agreed to locate here in consideration for such an inducement. Immediately upon their heels comes another offer, from an equally large concern, to move their works here upon the same terms. We doubt not that Elmira can become the largest manufacturing city in the United States, could she continue these munificent offerings. But, since it is wholly done by private citizens, from private purses, we suppose no one need complain about it, save those who subscribe. Elmira offers so many facilities for manufacturing purposes that it would seem that no other inducements are really necessary to have manufactories establish themselves with us. We have unsurpassed railroad facilities, are convenient to the hard and soft coal mines, our city an unusually healthy one, while our public schools are unsurpassed. No better point can be selected for manufacturing concerns of any kind. The iron furnaces and rolling mills furnish the kinds of iron usually employed in all sorts of manufactured implements. We are in communication with the Pennsylvania hardwood districts by means of three different railways. Three grand trunk lines, and three smaller railways afford ample shipping facilities. Land contiguous to these lines, and convenient to the city, can be purchased for manufactories at very reasonable rates. We offer a splendid opening for a heavy wagon manufactory and for the manufacture of agricultural implements. Our efficient board of trade will give all needed information to parties desiring to locate.

---

THE ANARCHISTS.—The New York Communists recently celebrated the anniversary of the Paris Commune of 1871, by a meeting in Concordia Hall. The attendance seems to have been very large, whereat we are astonished. Every one present wore a red ribbon, the officers being distinguished by red sashes. The walls were hung with revolutionary mottoes, that were draped in blood colors. Herr Most, the foreign blatherskite, made an address—more properly a harangue—in the course of which he said that "the Paris Commune was too humane, and that the commune of the future will be established regardless of humanity, and with a firm hand to wield the sword of destruction." It is a great pity that some Americanized foreigner, satisfied with his adopted country, did not apply the paddle of indignation to the person of this arrogant coward who seeks a peaceful country in which he attempts to stir up dissatisfaction and consequent laziness among the laboring classes. In permitting this sort of meetings, is not our country just a trifle *too* free? Is it generous toward the countries with which we are at peace to allow their communists to abide with us to stir up insurrections against their home authorities? If the contented workingmen of this country do not think it worth their while to give Herr Most a taste of his own philosophy, by mobbing him in every city where he attempts his traitorous harangues, then should the city authorities suppress the scoundrel for decency's sake.

---

ADVERTISING QUACKERY.—We are greatly surprised that such a reputable newspaper as the New York semi-weekly *Tribune*, should sell nearly an entire page of its advertising space [on Tuesday, December 26, 1882] to a lying and arrogant quack who advertises a cancer cure, which he claims to be infallible. To make matters worse, the advertisement is presented in the form of an interview, as though the editor had sought out this great curer of cancers, and had written him up for the benefit of the afflicted *Tribune* readers. The "interview" is crammed full of the most extravagant statements as to the wonderful cures performed by this great cancer doctor. It is stated that not a solitary instance of failure to cure the largest and most malignant cancer has ever occurred in his practice. The interviewer is shown immense cancers, from size of a tin cup to that of a half bushel, that have been "successfully removed" by the doctor—and numerous equally extravagant statements abound in this skillfully arranged advertisement.

Presented in the manner it is, under the seeming patronage of the *Tribune*, as though written by the editor himself, calling attention to a new discovery, etc., it is calculated to mislead many people, and leads us to the conclusion that the *Tribune* has descended from the high position it maintained when under the guidance of its eminent founder.

BANQUETING A MICROSCOPIST.—Prof. Joseph Leidy, the great microscopist, during a visit to Charleston, last winter, was given a reception and banquet by the prominent citizens of that city. Among the good things served upon that occasion was a peculiar fish, that always carries a tumor-like excrescence upon its tail. This rotundity is regarded by the natives as a great delicacy, so that the tails only of the fish that carry it are cooked and eaten. The Professor, not exactly pleased with the appearance of this delicate morsel, concluded to ascertain, on the following day, what his generous hosts were feeding him. He accordingly called at a fish market, selected a fish with a fine, fat tumor on his tail, and opened it. And what think you these Charleston seekers after delicacies had been banqueting on? Of course you can not guess, but we have the Professor's word for it, the delicate morsel—the lump—contained a tape worm.

---

AMENDED STATEMENT.—In the January number of this journal we commented upon the fine appearance and very capable work done on the proceedings of the American Society of Microscopists. In so doing we unintentionally did great injustice to Geo. E. Fell, M. D., F. R. M. S., who, we have since learned, performed a good share of the labor in preparing the transactions of the society for the press. We are glad to know that Prof. Kellicott had so capable an assistant, and hope Dr. Fell's aid will be retained in all future editions of that valuable work.

---

A MAGNIFICENT CABINET.—Dr. Lewis M. Eastman, of Baltimore, President of the Baltimore Microscopical Society, and known throughout the civilized world as a distinguished mounter of microscopic objects, possesses the largest cabinet in microscopy in the United States, if not in the world. Not only is his cabinet noted for its great extent, but also for the fineness of the collection. It is very full and complete in the departments of histology, pathology, microscopic botany and fungi, diatomaciæ, algæ, minerology, etc. His coal preparations are wonderfully fine, showing their vegetable cells in cross and longitudinal section, almost as distinctly as in a freshly mounted vegetable section. His double and triple stained vegetable and animal sections are marvels of neatness, surpassing any that we have heretofore seen. An entire week could easily be spent in looking at his collection of diatoms, alone; and such diatoms! Diatoms from all parts of the earth, selected and arranged slides, which, for cleanliness and beauty of arrangement, would make a diatomaniac wild with joy. We spent an entire afternoon looking at arranged diatoms without exhausting that department.

Baltimore boasts of two institutions for the visitation of scientists—Johns Hopkins University, the best equipped institution for biological research in this country, and Dr. Eastman's cabinet of microscopic objects—the finest and largest in the world.

---

"THE OLD DOCTOR."—Yes, that is what they call us now. Strange how suddenly senility struck us. It came not at an unexpected moment, but at an unguarded one. And this was the manner of its accomplishment:

"JEFFERSON MEDICAL COLLEGE,
PHILA., March 30, 1883.

"To 'Old' Dr. Up de Graff: Have just received notice that I have passed, at the head of my class.

"THAD S. UP DE GRAFF, JR.
"The young doctor."

We well remember our exultation and delight when the ordeal of passing the stern and dignified professors had been accomplished, and, having received our diploma, how self-satisfied we became, believing and confiding more then in our ability to cure the ills of humanity than we ever have since. How provoking it was, too, when patients, calling at the office, would pass us by and inquire for the "old doctor." That always effectually squelched us and made us wish for the gray hairs that would give us the appearance of knowing more than our patrons gave us credit for. Well, it has come at last. We are thoroughly installed as the "old doctor," of the fourth generation; and, although we may be forced to don spectacles, we give notice that our locomotive appendages still perform their required functions without aid of crutch or cane.

---

THAT SHAM FLOPPER.—Have you seen it? A device for removing an ornamental covering of the pillows, so that they may not be crammed under the bed or thrown in a heap in one corner of the room. Shams, you know, are strictly used to satisfy the æsthetic taste of the lady of the house, and are in no wise intended as a comfortable or desirable head-rest. Their fluted ruffles, starched surface, and heavily worked monograms in the center, would at once satisfy a sleepy but intelligent observer that it was constructed entirely for show. The lady visitor to your best room carefully folds the contrivances and lays them away where no injury can befall them. The man flings them upon the floor, or dives his greasy head into them, just to see how they behave. This latter treatment of them has always been a great annoyance to the good and patient housewife. So, a woman came to her relief and invented a neat little wire arrangement that folds the sham in the center and flops it above the head-board, well out of reach of head or hands. The woman who invented it deserves well of her sex. If she calls upon you, by all means give her an interview.

THE ASSASSIN DUKES.—Capt. Lyman Dukes, of Uniontown, Pa., who proclaimed that he had seduced the daughter of a gentleman, and who shot the father for fear that he justly would shoot first, was acquitted by the jury that tried him for murder. But his punishment is quite as severe as though he had been condemned to death, for he has been ordered by his enraged neighbors to leave the town, has been refused admittance to all the hotels in Harrisburg, is compelled to conceal himself wherever known, while the son and scandalized daughter are looking for him with loaded pistols. That chap had better been hung. It would have been less shocking to his nervous system—less harrassing than to be compelled to lock himself in a closet or jump from a back window every time a footstep is heard approaching his door. The daughter practices daily with a revolver, while a mild-eyed youth of nineteen —who can hit a quarter of a dollar at fifty yards—stands daily guard near his apartments, watching for him to come out upon the street. What a delightful state of mind the Captain must be in with such surroundings! Go hang yourself, old fellow—'tis the easiest way out of the difficulty.

AN ARRIVAL.—A large *amphiuma*, or "Congo snake," came to us by express, to-day, from the swamps of Louisiana, the gift of that celebrated microscopist, George C. Taylor, Esq., better known as "Old Gray Beard." Perched on the back of Mr. Amphiuma was an immense bullfrog that kept his excellency company all the way from New Orleans. They will both be sacrificed to the needs of science, in the preparation of objects for the microscope. The amphiuma is noted for affording the largest blood-disks known. It is therefore a very valuable specimen, particularly since it has traveled so far, and successfully resisted the cold weather.

THAT CODE OF MEDICAL ETHICS is giving some physicians and the American Medical Society a deal of trouble. About one year ago the Medical Society of the State of New York, by more than a two-thirds vote of that large body of medical gentlemen, agreed to amend their by-laws in such a manner as not to reflect upon themselves as gentlemen and to permit consultations—in the interests of humanity—with all legally qualified physicians. "Legally qualified," meaning all respectable physicians who had graduated at any recognized medical school. Of course this would "permit" a "regular" physician to consult with a homeopath—if the latter is willing. Great objection is made to this liberalism on the part of the regular fraternity, by a small minority who think such consultations undignified—but just why, have not sufficiently explained. In consequence of this action on the part of the New York society, the American society have threatened not to receive delegates from the former, and will therefore not recognize the New York physicians. What a terrible calamity! Just think of all of those great medical lights in New York city—Austin Flint, James Wood, Fordice Barker, and a hundred others that could be easily mentioned as being in the front rank of medical science—not being "recognized." Guess the New York physicians can stand it much better than the American society can afford to lose them.

KILLING MERCIFULLY.—Some people are forever concerning themselves about the comfort and well-being of animals at the very moment they had kicked a beggar for a meal from their kitchen door. The latest contrivance in this direction is the invention of Mr. George Law Fox, a London engineer, who desires mercifully to kill incurable and worn-out animals, particularly horses and dogs.

The animal to be killed is led into a stall with an iron floor, to which it is connected with the negative pole of a powerful condenser, of the capacity of one hundred microfards. The animal's head is wet with salt water, when the operator touches it with the positive pole and it falls dead. The death is believed to be painless, because physiologists have sufficiently demonstrated that a nervous vibration can not be communicated to the brain in less than a tenth of a second, and another tenth of a second must elapse before a sensation can result from the vibration thus communicated. One-fifth of a second must therefore elapse between the actual infliction of an injury, and the experience of pain from it.

It is for this reason that severe injuries are often inflicted upon workmen by rapidly revolving saws, without their being aware that the wound had been inflicted at the instant it was done. The cut was painless, because of the rapidity with which it was accomplished—before the nerves had time to report it to the brain. Recognizing this physiological principle, they adapt it to the killing of animals. A good device to be applied near the entrances of our houses, for the use of book agents and tramps.

---

THE BACILLUS.—It seems to have been abundantly proven by very capable pathologists and microscopists that many of the diseases prevalent among humanity are directly attributable to a minute living organism called *bacillus.* Different diseases are supposed to be the result of different forms of this organism —in other words, every disease has its own peculiar bacillus.

The disease to which sheep are subject, on the continent, known as *anthrax*, has been proven to be caused by a peculiar bacillus that invades the blood and tissues of the animal. But what is still more curious, these little animals are propagated in certain fluids, such as the cerum of the blood, sound animals inoculated therefrom when the disease becomes established in the inoculated creature. These bacilli are also "cultured" by producing several generations of them, inoculating a prepared fluid in which they are found to flourish, first from the diseased animal, then a fresh fluid from the last inoculation, and so on, until the culture is several generations remote from the original bacilli.

Now, if an animal is inoculated with this last culture, it is found to produce a mild form of the disease, that protects the animal from an attack of the more virulent one, just as we vaccinate with the cow-pox to cause a mild form of varioloid exempting the subject from small-pox.

Extensive experiments have been carried on in this line, vaccinations having been made with the bacilli of scarlet fever, typhoid fever, erysipelas, diphtheria, consumption, and more recently, whooping cough, with a view to preventing these highly contagious and dangerous diseases. As yet, experiments have been confined to animals, but we doubt not that sufficient knowledge will thus be obtained to warrant the inoculation of human beings for the prevention of the diseases enumerated, as well as for many others.

To show the results of the experiments of M. Pasteur in cultivating the bacillus of malignant charbon and inoculating sheep therewith, we have learned from the reports of the veterinary society of Eure-et-Loire, that the number of sheep vaccinated in this department was 79,312. Before vaccination the annual losses on these flocks amounted to 7,237, or about nine per cent; since vaccination, only 518 animals have perished, or about sixty-five one hundredth of one per cent. There were also vaccinated 4,652 cattle, among which the annual loss from charbon was 322. Since vaccination but eleven cows were lost.

This already sufficiently demonstrates the value of the discovery of, and experimentation with, the bacillus, for all of which we are indebted to the microscopists.

